# Colorectal cancer screening: Why immunochemical fecal occult blood tests may be the best option

**DOI:** 10.1186/1471-230X-12-183

**Published:** 2012-12-29

**Authors:** Kathy L Flitcroft, Les M Irwig, Stacy M Carter, Glenn P Salkeld, James A Gillespie

**Affiliations:** 1 Sydney School of Public Health, Edward Ford Building, A28 University of Sydney, 2006, Sydney, NSW, Australia

**Keywords:** Screening objectives, Colorectal cancer, Fecal occult blood testing, Harm minimization, Equity, Economic efficiency, Prevention, Participation

## Abstract

**Background:**

There are many test options available for colorectal cancer screening. The choice of test relates to the objectives of those offering or considering screening.

**Discussion:**

While all screening programs aim to detect disease early in order to improve the length and/or quality of life for the individual, some organizations and individuals prefer screening tests that offer the opportunity for cancer prevention. Others favor maximizing participation or the opportunity for shared decision-making, including discussion of information on test quality and availability. We propose three additional objectives for screening: minimizing harms, optimizing economic efficiency and maximizing equity of access to screening.

**Summary:**

Applying these objectives to colorectal cancer screening, we advocate the use of immunochemical FOBTs as the preferred screening strategy, as it satisfies all three of these important objectives.

## Background

There is a wide range of tests that may be offered to people considering colorectal cancer (CRC) screening. These include tests which allow for visual inspection of the bowel, such as colonoscopy and flexible sigmoidoscopy; imaging tests such as computed tomographic (CT) colonography, magnetic resonance (MR) colonography, capsule endoscopy and double contrast barium enema; and tests on fecal specimens such as fecal occult blood tests (FOBTs) and stool DNA tests. The choice of test depends to some extent on the preferences of the individuals or groups making these decisions: different screening objectives will favor different tests.

There are advantages and disadvantages to each test. For example, tests that use visual or imaging-based inspection of the colorectum involve bowel preparation that is unpleasant and potentially harmful. However, these tests do not have to be performed as often as tests that involve fecal sampling. Is it better to have a more burdensome test less often than a simple test more often? The answer depends in part on the implementation objectives of those offering the screening test and in part on the view of the individual considering the screening test.

All screening programs aim to detect disease early in order to improve the average length and/or quality of life for a population at risk of developing a particular disease. Any cancer screening program must satisfy this essential aim of reducing mortality and/or morbidity from the disease being screened for, and this article does not seek to downplay this fundamental criteria.

But all screening tests have the potential to cause harm. Within each screening program, there is also a range of other possible objectives – which can be thought of as additional objectives – that aim to increase the benefits of screening, or their distribution, in relation to the harms. Like the essential criterion of mortality/morbidity reduction, these additional objectives have moral value, and different people will prioritize them differently for different reasons.

For example, the US Multi-Society Task Force on Colorectal Cancer has placed a priority on cancer prevention. This has led to a preference for screening tests that are capable of detecting adenomatous polyps rather than just cancer, “as long as resources are available and patients are willing to undergo an invasive test” [1]. Preferred tests to satisfy this objective are flexible sigmoidoscopy every 5 years, colonoscopy every 10 years, double contrast barium enema every 5 years or CT colonography every 5 years.

In contrast, the focus of the US Preventive Services Task Force is on strategies that maximize the number of individuals who get screened, while emphasizing the need for shared decision-making [2]. Recommended options, based on these objectives, are annual screening with high-sensitivity fecal occult blood test, sigmoidoscopy every 5 years combined with high-sensitivity FOBT every 3 years, and screening colonoscopy every 10 years. While recommending that participants be given a choice among screening modalities, they do state that “colonoscopy and flexible sigmoidoscopy (to a lesser degree) entail possible serious complications” [2].

We recognize the validity of two of these objectives–preventing cancer and encouraging shared decision-making are both worthy aims, although they are potentially contradictory (if individuals do not wish to undergo invasive tests for initial screening). In contrast, we believe that maximizing participation should not be a primary aim of a screening program: not everyone will want to be screened, and high screening participation rates are not necessary to ensure cost-effectiveness [3]. The decision to be screened is dependent on an individual’s assessment of the relative benefits and downsides or harms for them personally.

This potential contradiction between encouraging shared decision-making and maximizing participation is well recognized [1,4-6]. This tension arises from differences in what is valued. If our objective is shared decision-making, we are valuing respect for individuals, expressed via an opportunity for people to meaningfully consider *whether they want to be screened*, and to receive services accordingly. Conversely, taking maximization of participation as a goal places value on improving efficiency or meeting targets. If maximizing participation is the goal, individuals should be actively encouraged to *be screened*, regardless of the personal consequences. But, unlike screening for infectious diseases, the benefits of cancer screening are no greater than the sum of benefits to each individual.

This article will not revisit these debates, but instead will focus on three additional objectives that we argue are equally worthy of consideration, not only for colorectal cancer screening programs, but potentially for any screening program, where a range of screening modalities exist.

## Discussion

This discussion first provides an overview of three additional objectives for screening, followed by an outline of how each of these objectives can be applied to CRC screening.

### Additional objectives

1) Minimization of harms.

In their seminal work on screening principles, Wilson and Jungner [7] were among the first to warn that population screening can cause harm to individuals. “In enthusiastically attacking disease at an early stage the Hippocratic principle … of primum non nocere [first do no harm] should not be neglected” [7]. Invariably, there is a trade-off between the benefits of early detection for a few individuals and the potential harms experienced by a much larger number of individuals within an asymptomatic screened population. This raises ethical concerns regarding whether the benefits outweigh the harms and how this can be determined. In fact, extensive screening procedures are still being advocated without evidence of their effectiveness, and despite their potential or actual capacity to cause harm (see for example Law [8] on prostate cancer screening and Wald [9] on CT body scanning). Harm minimization, defined as minimizing the risk of adverse consequences, is one important potential objective of screening.

2) Optimization of economic efficiency.

Given the competition for scarce health funding, and the potential opportunity costs involved in opting for population screening over primary prevention or disease treatment, it is crucial that screening is performed as efficiently as possible to maximize the return on investment. The purpose of screening average risk persons is not to diagnose a particular disease, but to identify those at higher risk of having this disease. The screening process is designed to limit the number of people who are invited for more expensive follow-up of the initial disease indicator. By separating the screening and diagnostic tests, the funding and resources required for follow-up are provided only to those most at risk of the disease, thus producing cost savings and optimizing economic efficiency, a second worthy objective of screening.

While all methods of CRC screening have been shown to be cost-effective, FOBT screening has consistently been reported to be highly cost-effective [10-13], so much so that government funders in several countries have established organized FOBT screening programs. Population screening with more expensive tests, even if they are performed less often, have not yet been publicly funded. However, following an RCT of flexible sigmoidoscopy in the UK, [14] which did not include a cost-effectiveness assessment, there are plans to add a one-off flexible sigmoidoscopy test to the existing FOBT National Health Service Bowel Cancer Screening Program for people in England aged 55 [15]. Those aged over 60 will continue to be offered biennial guaiac-based FOBT tests (gFOBTs) that are known to be less accurate, but are less expensive, than immunochemical FOBTs (iFOBTs).

The use of quantitative iFOBTs also has another important advantage. It allows for greater flexibility as screening program organizers can choose the cut-off point for a positive result, ensuring resource considerations and population preferences can be taken into account. This is particularly important when colonoscopy capacity is limited.

3) Maximization of equity of access.

Savings from redirection of resources away from diagnostic tests and towards screening tests whose focus is on identifying those in the population at higher risk of disease could be used to address inequities in access to screening, by allowing *all* those who stand to benefit from screening to access it if they choose to do so. More funding would become available to offer free screening to all those within the target population the evidence has identified as potentially benefiting more than harming.

### Applying these additional objectives to colorectal cancer screening

1) Minimization of harms.

There is high-level evidence about the benefits of screening for CRC using fecal occult blood tests. Meta-analysis of randomized controlled trial evidence of screening for CRC using gFOBTs showed a relative mortality risk reduction of 25% for those who attended at least one round of screening, compared with no screening [16]. This level of relative risk reduction has been considered significant enough by many first-world countries to justify the introduction of at least some limited form of CRC screening [17]. According to Church, the “best evidence to date does not support a large difference in effectiveness of the three main approaches to CRC screening (FOBT, flexible sigmoidoscopy and colonoscopy)” and so “the US approach of recommending all three screening strategies seems sound” [18]. However, basing screening strategies on the evidence of effectiveness alone downplays the importance of potential harms in determining the benefit-to-harm trade-off of screening.

Colonoscopy is widely regarded as the reference standard for CRC screening, but its use as a screening tool has the potential to cause considerable harms. Although colonoscopy will find most, but not all, cancers and advanced adenomas [1], it is an invasive procedure that may lead to severe bleeding (particularly if biopsy of polyps is performed as part of the procedure), perforation of the bowel and even death. Fortunately, these severe adverse events from colonoscopy are rare. A study published in 2008 estimated pooled rates of colonoscopy-related bleeding at 1.64/1,000, perforation at 0.85/1,000, and death at 0.074/1,000 [19]. Other downsides include the unpleasant bowel preparation, sedation that can lead to cardiopulmonary complications, and a colonoscopy miss rate for large adenomas of 6-12% and 5% for cancers [1]. The potential downsides of flexible sigmoidoscopy are fewer and less severe than those for colonoscopy, but are still present [2].

A harm minimization strategy favors a non-invasive screening test over an invasive one, so making FOBT preferable to colonoscopy or flexible sigmoidoscopy. While colonoscopy is necessary as a diagnostic test for colorectal cancer, it is not essential as a screening test for people at average risk. The purpose of screening average risk persons is not to diagnose, but to identify those at higher risk of having CRC. Rather than using invasive tests as the screening tool, and potentially subjecting all those being screened to serious harms, it seems more prudent to focus on detecting the strongest predictor of CRC – microscopic amounts of blood in feces – by using the safer and much less expensive iFOBT to identify those at greater risk of developing CRC. By doing so, the potential for serious harm from an invasive test will be limited to those who have the most to gain from screening, that is, those who have a positive FOBT and are at higher risk of developing CRC – those with a positive FOBT are 12-40 times more likely to have CRC than those with a negative test [20]. Furthermore, there is a significant body of evidence demonstrating the superior accuracy of iFOBTs over the older, less sensitive gFOBTs used in the original CRC screening trials [21-25]. Because iFOBTs are more accurate, use of these tests can reduce harms relative to gFOBT by reducing the number of people who are subjected unnecessarily to invasive investigations.

Recent data from Australia has shown one iFOBT has produced a positive predictive value (PPV) for cancer of 4.3%, for advanced adenomas of 23% and for non-advanced adenomas of 25%, giving an overall PPV for neoplastic lesions of 52.3% [26]–remarkably high given the test positivity rate was 7.7%. In other words, 3.3 cancers were found for every 1,000 people screened, and this figure would be much higher if screening covered the entire age range of 50-75 years, rather than just 50, 55 and 65 year olds currently offered screening in Australia, because the yield of significant pathology is higher in older people. Such a high level of sensitivity allows selective detection of advanced adenomas over non-advanced adenomas, and provides a means of focusing on the strongest predictor of CRC (fecal blood), while ignoring the much larger numbers of non-advanced adenomas that are not a direct threat to an individual’s health.

Another potential method to minimize harms is to further target the population for CRC screening by sex as well as age. For example, a recent study found the prevalence of advanced adenomas in men aged 45-49 years of age was comparable with women aged 55-59 years [27] raising the possibility of starting CRC screening later in women.

2) Optimization of economic efficiency.

Restricting colonoscopy to those at the highest risk of having CRC also makes sense from a resource allocation point of view, especially given the limitations resource constraints have been shown to have on full program implementation [28]. However, a large percentage of colonoscopies performed in the US and Australia do not follow expert guidelines. Goodwin et al. found that 46.2% of a large sample (24,071) of US Medicare patients who had a negative colonoscopy underwent a repeated examination in fewer than seven years, rather than the recommended 10 years, with 23.5% of the whole sample doing so with no clear clinical indication [29]. In Australia, over 440,000 colonoscopies were performed annually in 2005-06, with 75% of these undertaken in the private sector, and these figures are increasing by around five percent per year [30]. The significant growth in colonoscopies nationwide since 1995 suggests that colonoscopy is being used as a primary screening and surveillance tool [30], instead of iFOBTs as the Australian National Health & Medical Research Council (NHMRC) guidelines recommend. An alternative approach to invasive modes of CRC screening is supported by recent evidence from a systematic review [31] and modeling of CRC symptoms [32]. These studies identified population subgroups at higher risk of CRC, with increasing age, rectal bleeding and weight loss being the most accurate predictors of CRC. The systematic review concluded that, given the current evidence, “it seems wise to channel resources for cancer detection towards population based screening programs using FOBT rather than relying on identifying all cancers and precancerous polyps through investigating people with symptoms” [31]. Focusing on FOBT as the primary screening test can avoid unnecessary and wasteful use of resources that could then be redirected to other areas of the program. For example, if non-compliance with the NHMRC guidelines, and the resultant overly frequent and inappropriate use of colonoscopy, was addressed, it would free up colonoscopy capacity and enable the implementation of a full biennial iFOBT screening program for those aged over 50, as per the NHMRC guidelines, instead of the current limited program. Annual FOBT screening, where offered, could also be pared back to biennial, resulting in significant cost savings, while still delivering a relative risk reduction of up to 21% compared with a maximum estimate of 33% for annual screening as found in the Minnesota trial [33]. Such cost savings could then be put to worthwhile use within the CRC screening program.

3) Maximization of equity of access.

Savings from redirection of resources currently used for colonoscopy not recommended in the guidelines could be used not just to extend services, but to strategically address inequities in access to screening, by allowing *all* those who stand to benefit from screening to access it if they choose to do so. The opportunity costs from less efficient allocation of resources may not only result in adverse economic impacts, but also in undesirable ethical ones. Unless screening is available to all, regardless of a person’s ability to pay, those denied screening on economic grounds face potentially higher rates of CRC morbidity and mortality. If resources were redirected, more funding would become available to subsidize subsequent colonoscopy follow-up for those with positive tests, to support national advertising campaigns to increase awareness of CRC screening, to develop decision aids to help potential screenees assess the relative benefits and harms of screening based on their own values and risk, and to implement more targeted approaches to those populations that are less likely to participate. It is important to note that the objective of maximizing access to screening is not the same as maximizing participation. The primary concern should be offering a screening test that has been shown to be effective to all those who may benefit from it, while respecting their right to decline the test [5].

There is substantial evidence of inequitable participation in CRC screening programs. For example, in Australia, Indigenous Australians are significantly less likely to participate in CRC screening than non-Indigenous Australians [34], while in the UK, von Wagner and colleagues found reduced uptake of CRC screening in the most ethnically diverse and lower socioeconomic areas [35]. Levin has demonstrated the unequal distribution of reduced CRC mortality rates across the US which is strongly correlated with the uptake of screening [36]. The use of iFOBT by Kaiser Permanente in Northern California has contributed to a substantial increase in the CRC screening rate from 37% to 75% and in the Medicare population from 41% to 85% between 2005 and 2011 [36] − unprecedented levels of participation. Importantly, this increased screening rate is associated with earlier staging of CRC and a reduced incidence of CRC [36]. Such success has led Levin to comment that “The question facing clinicians and health care delivery system policy and decision makers is no longer whether to use FIT [fecal immunochemical test] as part of the screening menu of options, but which FIT to use and how to optimize FIT use” [36].

## Summary

Apart from the essential criterion of all cancer screening programs – reduction in mortality and morbidity – there is a range of potential additional objectives for screening programs including: prevention of disease, maximising participation, facilitating informed decision-making by screenees, minimising harms, optimising economic efficiency and maximising equity of access. In cases where there are several possible screening modalities, such as colorectal cancer screening, the preferred objectives will largely determine the choice of test. We have argued that because CRC screening with iFOBTs satisfies three important objectives – minimizing harms, optimizing economic efficiency and maximizing equity of access – it should be the preferred colorectal cancer screening strategy for countries with either opportunistic or nationally-organized CRC screening programs. Individuals should be initially offered iFOBT but, following informed discussion of all the options, may choose another colorectal cancer screening test, or choose not to be screened at all.

## Competing interests

All authors declare that they have no financial or non-financial competing interests.

## Authors’ contributions

KF, LI and GS conceived of the initial concept for this article. KF was primary author of the manuscript, but all authors made substantial contributions to earlier drafts and provided rigorous critique of subsequent versions. All authors have read and approved the final manuscript.

## Authors’ information

KF. Research fellow, Screening and Test Evaluation Program (STEP), Sydney School of Public Health, University of Sydney, Australia. LI. Professor of epidemiology, Screening and Test Evaluation Program (STEP), Sydney School of Public Health, University of Sydney, Australia. SM. Senior lecturer, Centre for Values, Ethics and the Law in Medicine and Sydney School of Public Health, University of Sydney, Australia. GS. Professor of health economics, Screening and Test Evaluation Program (STEP), Sydney School of Public Health, University of Sydney, Australia. JG. Associate professor, Sydney School of Public Health and Menzies Centre for Health Policy, Sydney School of Public Health, University of Sydney, Australia.

## Pre-publication history

The pre-publication history for this paper can be accessed here:

http://www.biomedcentral.com/1471-230X/12/183/prepub
